# Study on Gel–Resin Composite for Losting Circulation Control to Improve Plugging Effect in Fracture Formation

**DOI:** 10.3390/gels11080617

**Published:** 2025-08-07

**Authors:** Jinzhi Zhu, Tao Wang, Shaojun Zhang, Yingrui Bai, Guochuan Qin, Jingbin Yang

**Affiliations:** 1 Tarim Oilfield Branch, Korla 841000, China; zhujz147@163.com (J.Z.);; 2 R&D Center for Ultra Deep Complex Reservior Exploration and Development, China National Petroleum Corporation, Korla 841000, China; 3 Xinjiang Key Laboratory of Ultra-Deep Oil and Gas, Korla 841000, China; 4 Engineering Research Center for Ultra-Deep Complex Reservoir Exploration and Development, Korla 841000, China; 5 State Key Laboratory of Deep Oil and Gas, China University of Petroleum (East China), Qingdao 266580, China

**Keywords:** high-temperature- and high-pressure-resistant system, gel–resin, plugging system, drilling fluid loss, thermal stability, acid solubility

## Abstract

Lost circulation, a prevalent challenge in drilling engineering, poses significant risks including drilling fluid loss, wellbore instability, and environmental contamination. Conventional plugging materials often exhibit an inadequate performance under high-temperature, high-pressure (HTHP), and complex formation conditions. To address that, this study developed a high-performance gel–resin composite plugging material resistant to HTHP environments. By optimizing the formulation of bisphenol-A epoxy resin (20%), hexamethylenetetramine (3%), and hydroxyethyl cellulose (1%), and incorporating fillers such as nano-silica and walnut shell particles, a controllable high-strength plugging system was constructed. Fourier-transform infrared spectroscopy (FTIR) and thermogravimetric analysis (TGA) confirmed the structural stability of the resin, with an initial decomposition temperature of 220 °C and a compressive strength retention of 14.4 MPa after 45 days of aging at 140 °C. Rheological tests revealed shear-thinning behavior (initial viscosity: 300–350 mPa·s), with viscosity increasing marginally to 51 mPa·s after 10 h of stirring at ambient temperature, demonstrating superior pumpability. Experimental results indicated excellent adaptability of the system to drilling fluid contamination (compressive strength: 5.04 MPa at 20% dosage), high salinity (formation water salinity: 166.5 g/L), and elevated temperatures (140 °C). In pressure-bearing plugging tests, the resin achieved a breakthrough pressure of 15.19 MPa in wedge-shaped fractures (inlet: 7 mm/outlet: 5 mm) and a sand-packed tube sealing pressure of 11.25 MPa. Acid solubility tests further demonstrated outstanding degradability, with a 97.69% degradation rate after 24 h in 15% hydrochloric acid at 140 °C. This study provides an efficient, stable, and environmentally friendly solution for mitigating drilling fluid loss in complex formations, exhibiting significant potential for engineering applications.

## 1. Introduction

Well leakage refers to the phenomenon where drilling fluid leaks from the wellbore to the surrounding formation during the drilling process, causing formation damage. This is one of the common complex situations in drilling engineering. If not dealt with in time, it may lead to serious engineering problems and safety hazards [1,2,3]. Its incidence rate is influenced by various factors, including geological conditions (such as faults and fracture development), formation pressure, and drilling fluid performance [4,5,6]. At present, the incidence rate of well leakage in global drilling is high, accounting for about 20% to 25% of the total drilling [7,8,9]. In drilling operations, once a well leakage occurs, it will lead to a large loss of drilling fluid, increase the cost of drilling fluid replenishment, and at the same time, reduce drilling efficiency and prolong the drilling time, resulting in huge economic losses [10,11,12]. CT technology proves that drilling fluid loss may cause the wellbore to lose support, triggering wellbore collapse, stuck pipe accidents, and even an increased risk of well blowout, posing a serious threat to downhole safety [13,14,15,16]. In addition, drilling fluid invasion into the formation may pollute the reservoir, affecting subsequent oil and gas extraction, and may also expand fractures, increasing the difficulty of plugging leaks [17]. Well leakage will also lead to frequent adjustment of drilling parameters, increase the complexity of operation, and even require the optimization of the wellbore trajectory to avoid high-risk formations, which will have many adverse effects on subsequent operations [18,19,20].

In the field of oil and gas drilling, there are numerous traditional drilling fluid plugging agents and plugging materials internationally. The most common ones are bridging materials (such as calcium carbonate, fruit shell fibers, and mica) and high-water-loss materials (such as polymers, diatomaceous earth, and cement) [21,22]. In order to better cope with the problem of well leakage, some researchers have tried to develop other new plugging materials. Soft and flexible plugging materials (such as elastic graphite, modified polypropylene fibers) can deform and seal according to the characteristics of the leakage point [23,24]. Polymer gel plugging materials adapt to cracks and seal them by forming a three-dimensional network structure. Yang’s team developed a self-healing amphoteric polymer–silica nanoparticle microgel made of 4-Styrenesulfonic acid sodium salt and 3-methacrylamido-N,N,N-trimethylpropan-1-aminium chloride. Within the temperature range of 80–150 °C, the microgel exhibits a good plugging performance, and it can maintain a stable plugging effect even at a high temperature of 150 °C [25]. Water-absorbing and swelling materials expand and fill cracks by absorbing liquids. Zhang and others prepared and characterized submicron polymer microspheres as a plugging agent. In the core plugging experiment, after adding 3% polymer microspheres, the permeability of the core was reduced by 96%, showing an excellent plugging effect [26]. Composite plugging materials can leverage their advantages through the synergistic effect of various materials. Cui et al. prepared a carboxylated graphene oxide–silica composite material through a two-step method, and the composite material showed a better plugging performance than silica and carboxylated graphene oxide alone in pressure transmission tests and permeability tests [27]. Reza Lashkari synthesized an intelligent leakage plugging material with shape memory polyurethane [28]. When the concentration of this plugging agent reaches 114 kg/m^3^, it can withstand a sealing pressure of up to 100 bar in simulated cracks, and the cumulative loss in this pressure is only 72 cm^3^. The selection of these materials needs to be considered comprehensively according to geological conditions, crack size, and the compatibility of drilling fluid systems, in order to achieve the best leakage plugging effect.

High-intensity curing resins can solidify in cracks and play a role in plugging leaks by forming a dense cross-network structure. Thermosetting resins, with their excellent mechanical properties, thermal stability, chemical stability, and electrical insulation properties, account for as much as 15% of global synthetic resins. Common thermosetting resins include epoxy resin (EP), phenolic resin (PF), unsaturated polyester resin (UPR), etc. [29,30]. Khawlah Alanqari and others studied and designed a new epoxy resin formulation, which contains an epoxy resin and a chemical activator. It can undergo a polymerization reaction without adding water and can successfully girlify and coagulate after fluid enters the wellbore [31]. Qiu’s team prepared microcapsules using gelatin, arabic gum, and epoxy resin. These microcapsules, together with inert particles of calcium carbonate, are used as a composite plugging material to achieve physical–chemical synergistic plugging of formation fractures. After adding microcapsules, the plugging area formed by the resin system is made more compact, and the maximum plugging pressure increases by more than 22.2%. The drilling fluid loss is reduced by more than 50.8% [32]. Li and others prepared a self-repairing resin plugging material using epoxy resin, succinic anhydride, and polyethylene glycol as raw materials. Its temperature resistance reaches 180 °C, and it has good mechanical strength and elasticity. Its pressure-bearing capacity for a 5×4 mm crack can reach 10 MPa [33]. Zhao synthesized a shape memory epoxy resin for oil-based drilling fluids, whose glass transition temperature range is between 94 and 122 °C, and the material expansion rate can reach 74%. It can withstand a pressure of 15 MPa, effectively seal cracks under high-temperature conditions, reduce drilling fluid leakage, and improve drilling efficiency and safety [34]. Zhu and others developed a resin–gel–rubber expandable sealing particle based on sodium polyacrylate graft modification of starch. Under 90 °C conditions, its expansion rate rapidly increased to 24% within 6 h. The expanded sealing particles have an excellent toughness and elastic deformation ability, showing a compressive strength of 7 MPa and demonstrating an excellent crack sealing performance [35]. Therefore, the application space of thermosetting resin in the field of drilling fluid leakage plugging is vast, and it is an important technical means to solve complex leakage problems.

In this study, we constructed a controllable high-intensity anti-high-temperature condensation gel–resin plugging system for drilling fluid leakage problems. We optimized conditions such as the concentration of the resin matrix and the ratio of the curing agent, and we explored the influence of factors such as drilling fluid mixing, drilling fluid pollutants, formation water, gel polymer isolation fluid, and mineralization on the condensation of the plugging system. We studied the high-temperature stability, thickening performance, and residence performance of the resin under downhole conditions, and we evaluated the pressure plugging ability of the gel–resin condensation plugging system under complex conditions.

## 2. Results and Discussion

### 2.1. Preparation and Characterization

#### 2.1.1. The Optimization of the Formulation of the Gel–Resin Composite

**Figure 1:**
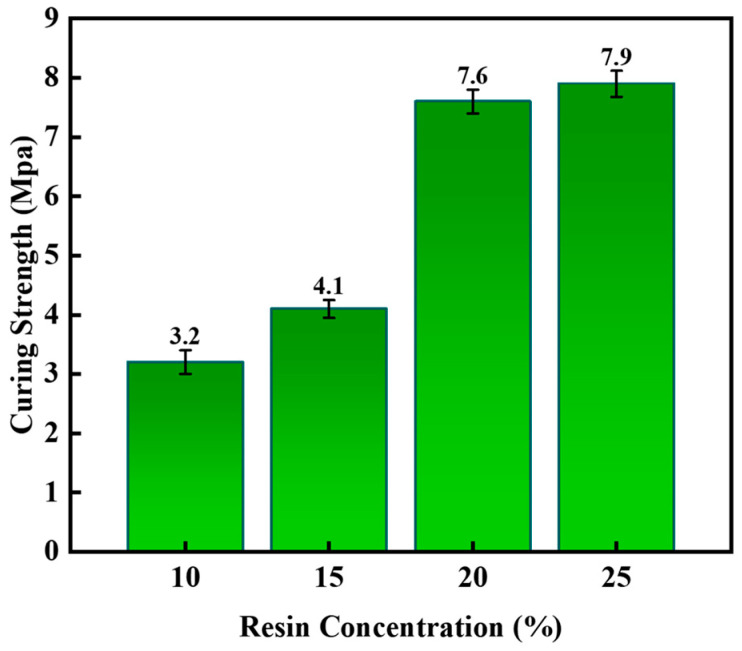
The influence of the resin concentration on the curing pressure of the system.

The formulation ratio of the gel–resin composite material can effectively improve the rheological properties, coagulation strength, coagulation temperature, and compressive bearing capacity of the gel–resin composite. In order to obtain an excellent leakage plugging material, the concentration of epoxy resin, hexamethylene tetramine, and hydroxyethyl cellulose was optimized. Set up experimental groups with the proportions of bisphenol A epoxy resin at 10%, 15%, 20%, and 25%, respectively, add 3% hexamethylene tetramine, 1% hydroxyethyl cellulose, 0.5% diethylenetriamine, and 10% filler (4% nano-silica + 2% walnut shell + 4% quartz sand) in turn, and mix them thoroughly. Place the mold in an oven set at a certain temperature for the condensation reaction. The condensation temperature is set at 140 °C, and the condensation time is 150 min. Observe the condensation effect. The curing strength (uniaxial compressive strength) shown in Figure 1 is the most suitable, and the coagulation effect of the leakage-stopping material with a concentration of 20% of bisphenol A epoxy resin.

**Figure 2:**
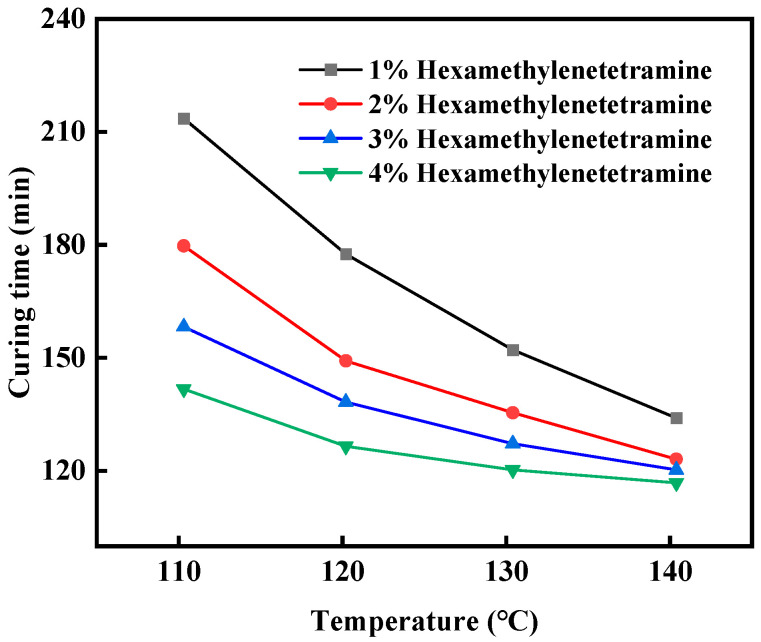
The coagulation effect of gel–resin under different concentrations of hexamethylene tetramine.

Set up experimental groups with the proportions of hexamethylene tetramine at 1%, 2%, 3%, and 4%, respectively, add 20% bisphenol A epoxy resin, 1% hydroxyethyl cellulose, 0.5% diethylene triamine, and 10% filler (4% nano-silica + 2% walnut shell + 4% quartz sand) in turn, and mix them thoroughly. Place the mold in an oven set at a specific temperature for the condensation reaction. Set the condensation temperatures at 110 °C, 120 °C, 130 °C, and 140 °C and observe the condensation effect. As shown in Figure 2, the coagulation effect of the leakage plugging material with a concentration of 3% hexamethylene tetramine is the most suitable.

**Figure 3:**
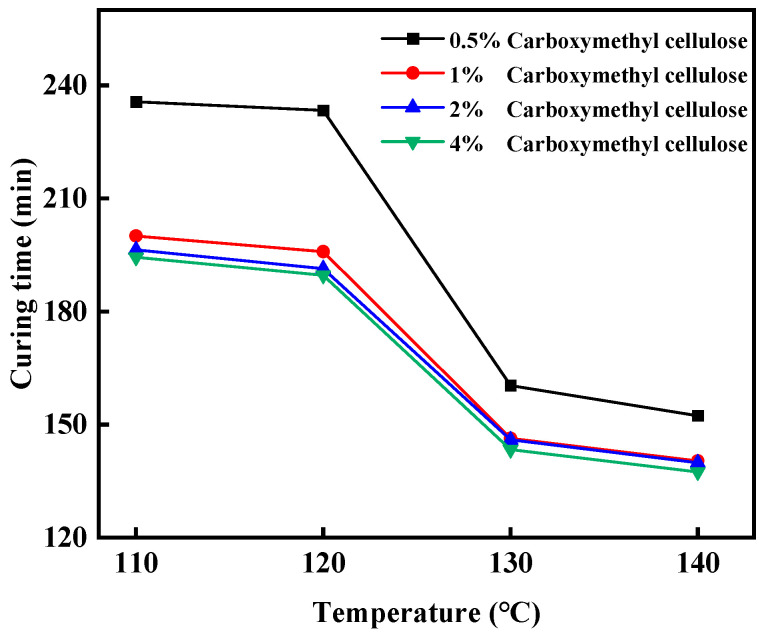
The coagulation effect of gel–resin under different concentrations of sodium carboxymethylcellulose.

Set up experimental groups with hydroxyethyl cellulose proportions of 0.5%, 1%, 2%, and 4%, respectively, add 20% bisphenol A epoxy resin, 3% hexamethylene tetramine, 0.5% diethylene triamine, and 10% filler (4% nano-silica + 2% walnut shell + 4% quartz sand) in turn, and mix them thoroughly. Place the mold in an oven set at a specific temperature for the condensation reaction. The condensation temperatures are set at 110 °C, 120 °C, 130 °C, and 140 °C, and the condensation time is 150 min. Observe the condensation effect. As shown in Figure 3, the coagulation effect of the leakage plugging material with a hydroxyethyl cellulose concentration of 1% is the most suitable.

#### 2.1.2. Characterization of the Gel–Resin Composite

**Figure 4:**
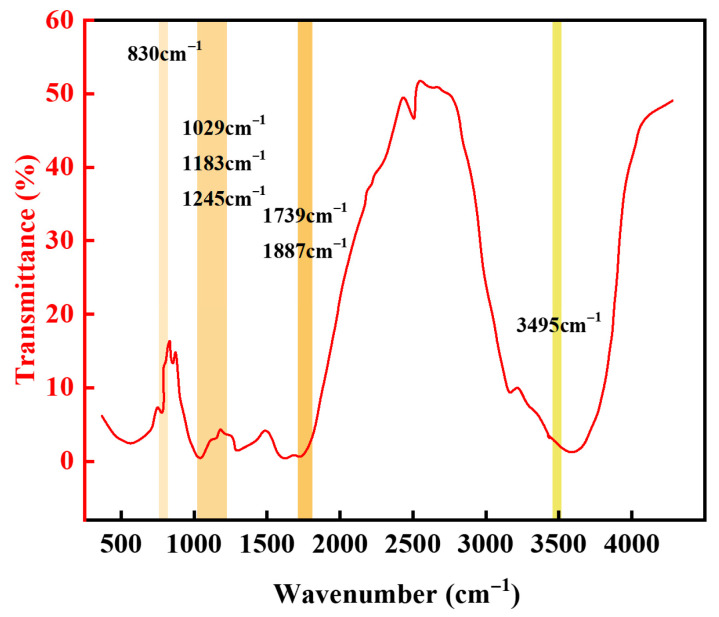
Infrared characterization results of lost circulation material.

As shown in Figure 4, the infrared spectroscopy analysis indicates that the characteristic peaks of the resin exhibit a regular evolution. In the infrared spectrum, the broad peak at 3495 cm^−1^ is the stretching vibration peak of hydroxyl groups, indicating that hydroxyl groups participate in the reaction and some hydroxyl groups are consumed. The characteristic peak intensity of epoxy groups at 830 cm^−1^ is significantly weakened, indicating that epoxy groups fully participate in the ring-opening reaction. The 1887 cm^−1^ and 1739 cm^−1^ peaks belong to the characteristic absorption peaks of C=O in acid anhydrides and the stretching vibration peaks of C=O in ester groups. The appearance of the 1739 cm^−1^ peak indicates the formation of ester structures after curing reaction. The peaks at 1245 cm^−1^, 1183 cm^−1^, and 1029 cm^−1^ belong to the antisymmetric and symmetric stretching vibration peaks of C-O-C [36]. This series of spectral feature changes effectively verifies the successful synthesis of the target gel–resin composite.

**Figure 5:**
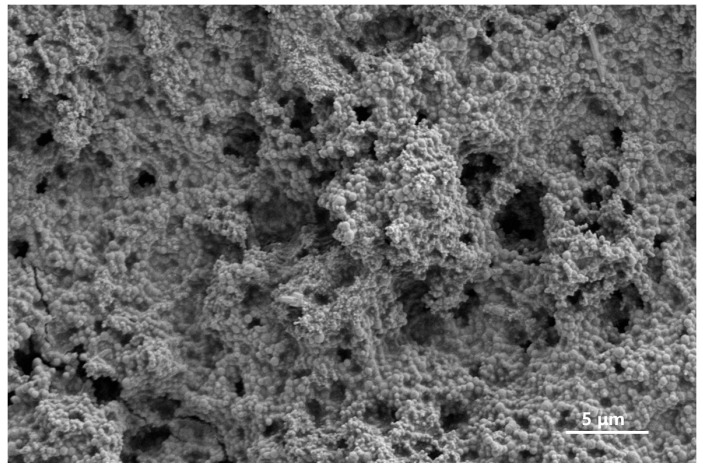
Result of SEM characterization of gel–resin system.

Scanning electron microscopy was used to observe the microstructure of the gel–resin coagulation system, as shown in Figure 5. The gel–resin coagulation system can form a chain or three-dimension network structure after curing. On the one hand, epoxy resin and curing agents and other chemical agents form a gel–resin main structure through covalent bonds such as ether bonds. On the other hand, fillers such as silica and quartz sand combine with the system through non-covalent interactions like hydrogen bonds, altering the surface morphology of the gel–resin system.

**Figure 6:**
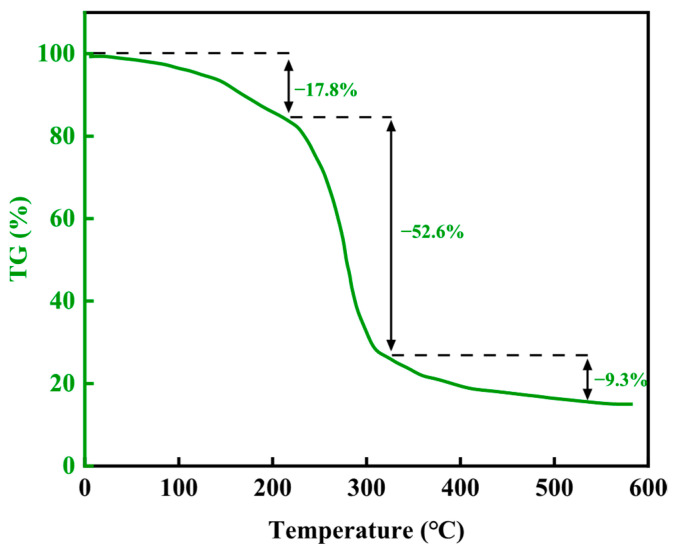
Thermogravimetric analysis results of the lost circulation material.

The thermogravimetric curve is shown in Figure 6. By analyzing the mass loss of the material in different temperature ranges, the thermal stability characteristics of chemical bonds in the molecular structure can be effectively evaluated. The thermogravimetric analysis of this resin plugging system is divided into three stages. The initial decomposition temperature is about 220 °C, and due to the loss of bound water and free water, the material loses about 17.8% of its mass. The second stage is from 220 °C to 322 °C, and the mass loss in this stage is about 52.6%. The last stage is from 322 °C to 543 °C, and the structure of the resin plugging agent further decomposes, with a moderate loss of about 9.3%. Therefore, this material has good thermal stability before 220 °C.

### 2.2. The Rheological Properties of the Gel–Resin Composite Coagulation System

**Figure 7:**
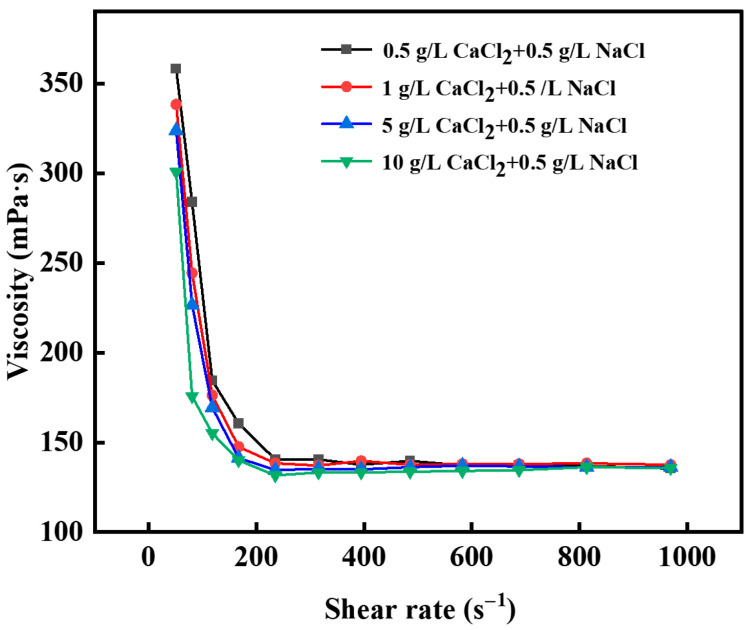
The change in viscosity of the condensation system with the shear rate at room temperature.

Viscosity is an important parameter for characterizing rheological properties. Good fluidity and pumpability of materials ensure that they can be smoothly injected into cracks or pores during the construction process. As shown in Figure 7, the viscosity of the composite gel–resin solution tested by the Haake rheometer before coagulation changes with the shear rate. At room temperature (25 °C), the initial viscosity of the coagulation system of the gel–resin composite is between 300 and 350 mPa·s, and the viscosity gradually decreases with the increase in the shear rate, and finally tends to stabilize; the gel–resin composite system has the “shear thinning” property, which is conducive to on-site pumping and means there is no need to use a high-pressure pump truck for pumping. This is consistent with the situation reported in the literature [37].

We tested the rheology of the coagulation system of the gel–resin composite under room temperature and different stirring times, and we simulated the solution state of the system during the ground slurry mixing process. The results are shown in Table 1. The results show that the apparent viscosity of the controllable coagulation system is 46.5 mPa·s at room temperature; after stirring for 5 h, the apparent viscosity is 49.5 mPa·s; and after stirring for 10 h, it is 51 mPa·s.

Among them, φ_600_ refers to the reading of the 6-speed rotational viscometer at a speed of 600 RPM; the initial cutting force is the maximum reading obtained by allowing the liquid to stand for 10 s after stirring and rotating at 3 RPM; the final cutting force is the maximum reading obtained by allowing the liquid to stand for 10 min after stirring and rotating at 3 RPM; the calculation method for apparent viscosity (AV) is φ_600_/2; the calculation method for plastic viscosity (PV) is φ_600_ − φ_300_; the calculation method for dynamic shear force (YP) is 2 × φ_300_ − φ_600_; and the unit is mPa·s.

**Figure 8:**
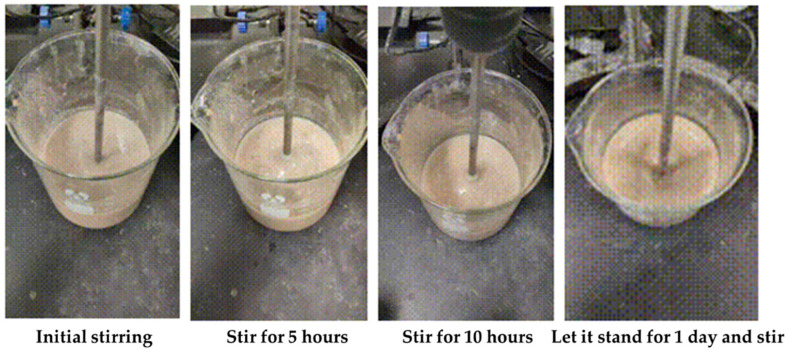
Images of the gel–resin composite coagulation system after stirring for different times under room temperature conditions.

Under room temperature conditions, the apparent viscosity of the gel–resin composite system is relatively stable, and there is no thickening, so it will not coagulate in the slurry mixing tank, as shown in Figure 8.

### 2.3. Factors Affecting Coagulation Performance of Gel–Resin Composite System

#### 2.3.1. Effect of Drilling Fluid Mix on Coagulation System of Gel–Resin Complex

**Figure 9:**
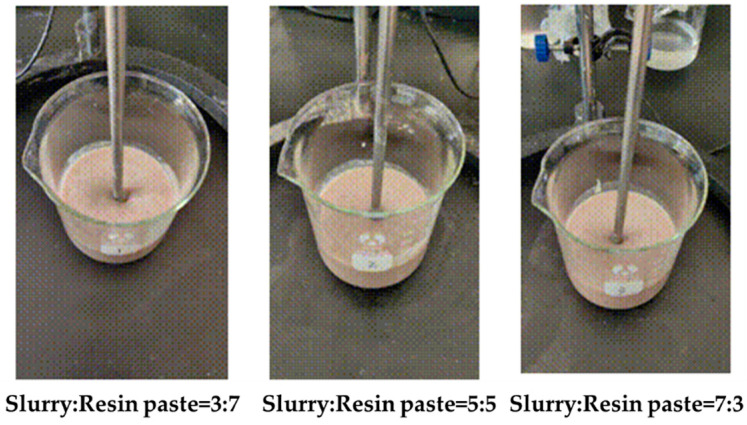
Images of different drilling fluid mixing systems with different additions.

Mix the bentonite drilling fluid and gel resin slurry in the proportions of 3:7, 5:5, and 7:3 to study the effect of the drilling fluid dosage on the rheology of the gel–resin complex coagulation system, as shown in Figure 9. The addition of drilling fluid will have a certain impact on the rheology of the gel–resin composite coagulation system. The apparent viscosity of the gel–resin composite system itself is 90 mPa·s at room temperature, and after adding drilling fluid in 3:7, 5:5, and 7:3 ratios, the apparent viscosity slightly increases due to the hydration effect, to 92.5 mPa·s, 102.5 mPa·s, and 103.5 mPa·s, respectively. The experimental results are shown in Table 2.

**Figure 10:**
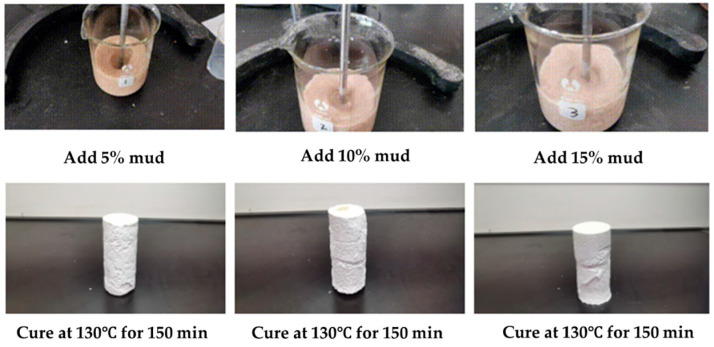
Images of coagulation effect of the gel–resin slurry system.

In order to further study the influence of the addition of bentonite drilling fluid on the coagulation effect of the gel–resin composite system, we sequentially added 5%, 10%, and 15% bentonite drilling fluid to the gel–resin composite system, and we observed its coagulation effect under the conditions of 130 °C and hot rolling for 150 min. This study shows that the addition of a small amount of bentonite drilling fluid has a minimal impact on the coagulation strength of the gel–resin composite system (Figure 10). Therefore, during the process of on-site slurry preparation, the part of drilling fluid remaining in the slurry preparation tank will affect its coagulation effect, so before preparing slurry, we should clean the slurry preparation tank; alternatively, we should use a clean water tank to prepare it.

**Figure 11:**
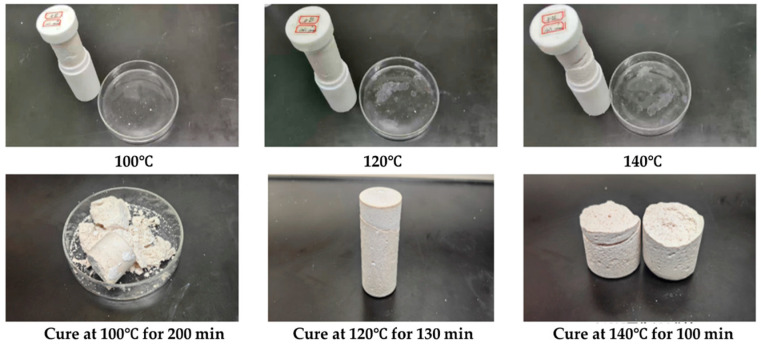
Images of coagulation effect of the gel–resin composite system prepared by well fluid.

We tested the coagulation effect of a gel–resin composite system prepared with well mud instead of water at 100 °C, 120 °C, and 140 °C, as shown in Figure 11. The experimental results showed that the gel–resin composite system prepared with well mud could form a good coagulation body at 100 °C for 200 min, 120 °C for 130 min, and 140 °C for 100 min, but the samples showed brittleness, and there were different degrees of fracture phenomena.

**Figure 12:**
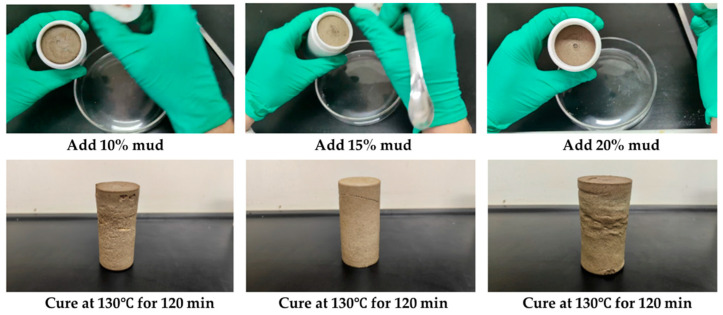
Images of coagulation effect of KCl polysulfonate drilling fluid on the gel–resin composite system.

At the same time, we studied the coagulation and plugging effect of the resin coagulation plugging system after adding 10%, 15%, and 20% KCl polysulfonate drilling fluid successively and rolling it at 130 °C for 150 min, as shown in Figure 12. The results show that the gel–resin composite system of 10% and 15% KCl polysulfonate drilling fluid can still coagulate into a high-strength coagulation body, and the coagulation strength is slightly reduced under the addition of 20% KCl polysulfonate drilling fluid.

In the resin coagulation plugging system, 10%, 15%, and 20% of the KCl polymeric drilling fluid system were sequentially added, and it was heated and rolled for 150 min at 130 °C to coagulate into a resin coagulate. The compressive strength of the samples after coagulation was tested. The experimental results are shown in Table 3. The compressive strengths of the samples where we added 10%, 15%, and 20% of the KCl polymeric drilling fluid system were 6.36 MPa, 5.75 MPa, and 4.29 MPa, respectively.

#### 2.3.2. The Influence of Drilling Fluid Pollution on the Gel–Resin Composite System

**Figure 13:**
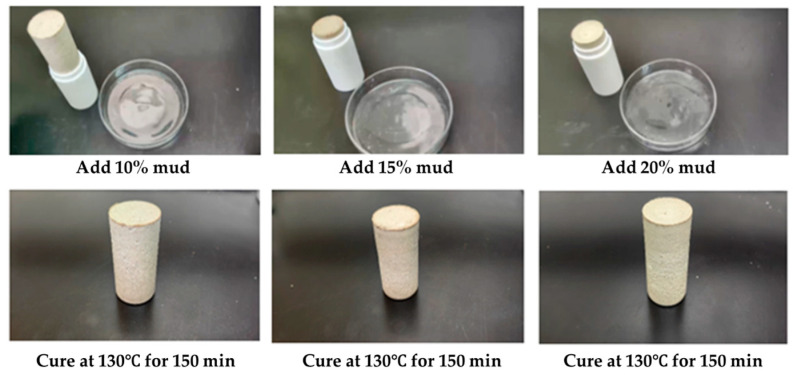
Images of influence of drilling fluid of Tarim on the coagulation effect of the gel–resin coagulation system.

The anti-pollution performance of the gel–resin coagulation plugging system was studied using the Tarim drilling fluid system, and its formula is shown in Table 4. In the resin coagulation plugging system, 10%, 15%, and 20% of the Tarim drilling fluid system were added in turn, and it was heated and rolled at 130 °C for 150 min (Figure 13). After mixing different amounts of the Tarim drilling fluid system with the gel–resin composite system, a coagulation body with certain strength could be formed, but the strength was lower than that of the coagulation body prepared with clean water.

In the resin coagulation plugging system, 10%, 15%, and 20% of the Tarim drilling fluid system were added sequentially, and it was heated and rolled for 150 min at 130 °C. The compressive strength of the samples after coagulation was tested. The experimental results are shown in Table 5. The compressive strengths of the drilling fluid with 10%, 15%, and 20% of the Tarim drilling fluid system were 7.39 MPa, 6.14 MPa, and 5.04 MPa, respectively. Therefore, the drilling fluid remaining in the mixing tank will affect its coagulation effect, and it is necessary to do a good job of cleaning the tank before mixing.

#### 2.3.3. The Influence of Formation Water on the Gel–Resin Coagulation System

**Figure 14:**
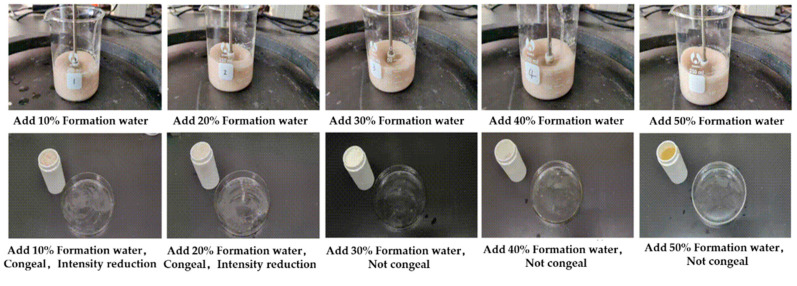
Images of influence of adding different amounts of formation water on the gel–resin coagulation system.

We added 10%, 20%, 30%, 40%, and 50% Tarim formation water (mineralization degree 166.5 g/L), respectively, to the gel–resin composite system to mix the slurry, and the dilution resistance of the gel–resin composite system after mixing with formation water was determined. The research results revealed (as shown in Figure 14) that adding 10–20% Tarim formation water to the gel–resin composite system has a certain dilution effect, but the system can still coagulate, and the coagulation strength is reduced; adding 30–50% Tarim formation water to the gel–resin composite system has a greater dilution effect. With the increase in the addition amount, the system coagulates poorly or even fails to coagulate at all.

**Figure 15:**
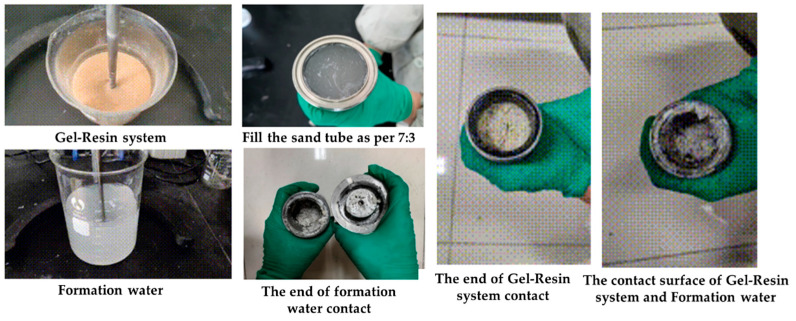
Images of 7:3 volume ratio of gel–resin composite system and formation water and its influence on the resin coagulation system.

The gel–resin composite system and the Tarim formation water were loaded into the sand-filled tube at a volume ratio of 7:3. The gelation effect at the contact surface between the gel–resin composite system and the formation water was observed for 3 h at 120 °C. The research results demonstrated (as shown in Figure 15) that under the condition of a 7:3 volume ratio, there will be a length at the contact surface where the gelation effect is slightly worse, and gelation can still be achieved far from the contact surface. When the gel–resin composite system is pumped and injected in the field, it is necessary to first pump and inject the spacer liquid plug to prevent the formation water from diluting the resin mortar system.

**Figure 16:**
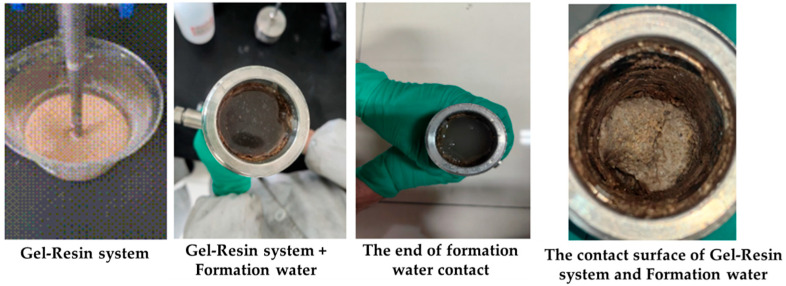
Images of the 5:5 volume ratio of gel–resin composite system and formation water and its influence on the resin coagulation system.

The gel–resin composite system and the Tarim formation water were loaded into the sand-filled tube in a 5:5 volume ratio and condensed for 3 h at 120 °C. The condensation effect at the contact surface between the gel–resin composite system and the formation water was observed. The research results demonstrated (as shown in Figure 16) that under the condition of a 5:5 volume ratio, the condensation effect at the contact surface is significantly affected, and the condensation effect of the entire gel–resin composite system slug is significantly reduced. Therefore, when pumping the gel–resin composite system on-site, it is necessary to first pump the spacer liquid slug to prevent the formation water from diluting the gel–resin composite system.

#### 2.3.4. The Influence of Polymer Gel Isolation Fluid on the Gel–Resin Composite System

**Figure 17:**
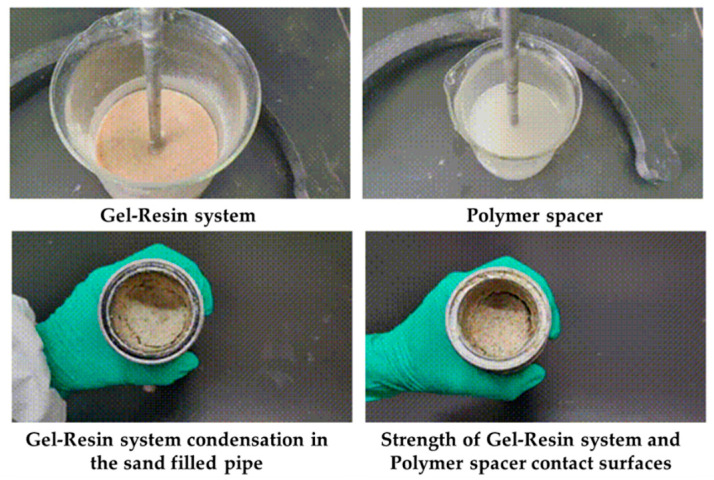
Images of the 7:3 volume ratio of gel–resin composite system and polymer isolation fluid and its influence on the resin coagulation system.

Load the gel–resin composite system and the polymer isolation fluid into the sand-filled tube at a volume ratio of 7:3 and observe the coagulation effect at the contact surface between the gel–resin composite system and the polymer isolation fluid under 120 °C for 3 h. The research results are shown in Figure 17. Under the condition of a 7:3 volume ratio, the gel–resin composite system can coagulate and form a coagulate well under the protection of the polymer isolation fluid, and the coagulation strength is high. Therefore, when the gel–resin composite system is pumped in the field, a certain volume of polymer spacer fluid plug can prevent the dilution effect of formation water.

**Figure 18:**
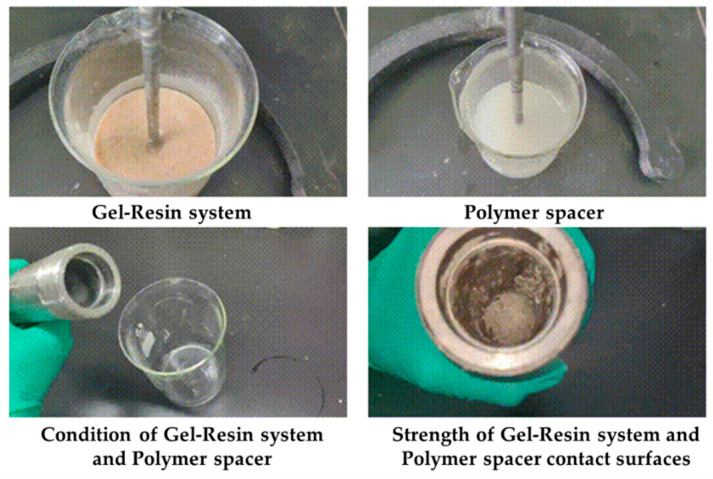
Images of the 5:5 volume ratio of gel–resin composite system and polymer isolation fluid and its influence on the resin coagulation and leakage plugging system.

The gel–resin composite system and the polymer isolation fluid were loaded into the sand-filled tube in a 5:5 volume ratio and solidified at 120 °C for 3 h. The solidification effect at the contact surface between the gel–resin composite system and the polymer isolation fluid was observed. The research results are shown in Figure 18. Under the condition of a 5:5 volume ratio, the gel–resin composite system can solidify and form a solidified body under the protection of the polymer isolation fluid, but its strength is relatively low. Therefore, when the gel–resin composite system is pumped on-site, it is beneficial to prevent the impact of formation water dilution on the solidification effect of the gel–resin composite system by pumping a plug of spacer fluid.

**Figure 19:**
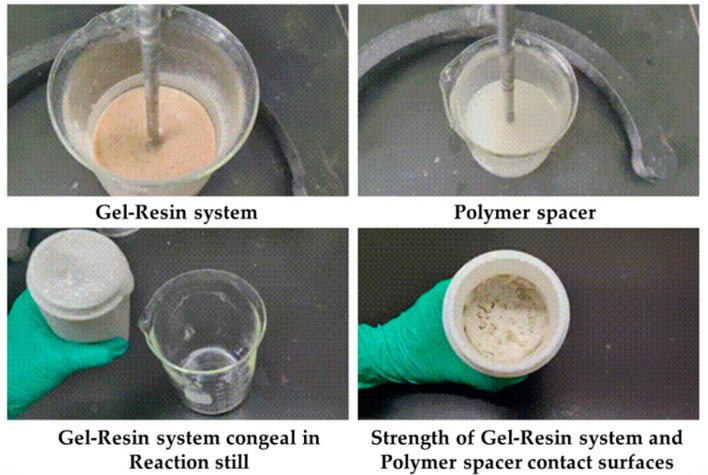
Images of the 7:3 volume ratio of the gel–resin composite system and polymer isolation fluid and its influence on the resin coagulation and leakage plugging system.

Load the gel–resin composite system and the polymer isolation fluid into the reactor at a volume ratio of 7:3 and condense them at 120 °C for 3 h, observing the condensation effect at the contact surface between the gel–resin composite system and the polymer isolation fluid. The research results are shown in Figure 19. Under the condition of a 7:3 volume ratio, the gel–resin composite system can form a high-strength condensate in a sealed reactor under the protection of the polymer isolation fluid. Therefore, a small amount of polymer spacer fluid plug can prevent the influence of formation water dilution on the condensation effect of the gel–resin composite system.

**Figure 20:**
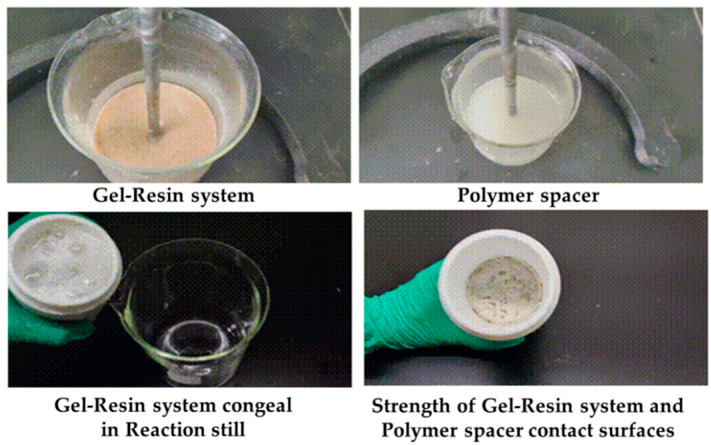
Images of the gel–resin composite system and the polymer isolation fluid in a 5:5 volume ratio and its influence on the resin coagulation and leakage plugging system.

The gel–resin composite system and the polymer isolation fluid were loaded into the reactor in a 5:5 volume ratio and condensed at 120 °C for 3 h, with us observing the condensation effect at the contact surface between the gel–resin composite system and the polymer isolation fluid. The research results are shown in Figure 20. Under the condition of a 5:5 volume ratio, the gel–resin composite system can form a high-strength condensate in a sealed reactor under the protection of the polymer isolation fluid. Therefore, when the gel–resin composite system is pumped into the spot, it is beneficial to prevent the influence of formation water dilution on the condensation effect of the gel–resin composite system by pumping the spacer fluid plug.

#### 2.3.5. The Influence of Mineralization on the Coagulation Effect of the Gel–Resin System

**Figure 21:**
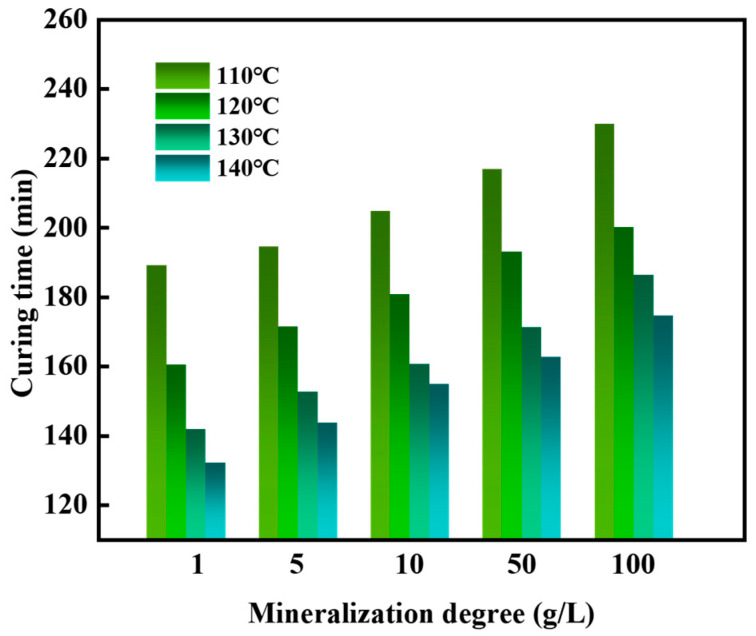
Influence of mineralization on the setting time of gel–resin slurry system.

**Figure 22:**
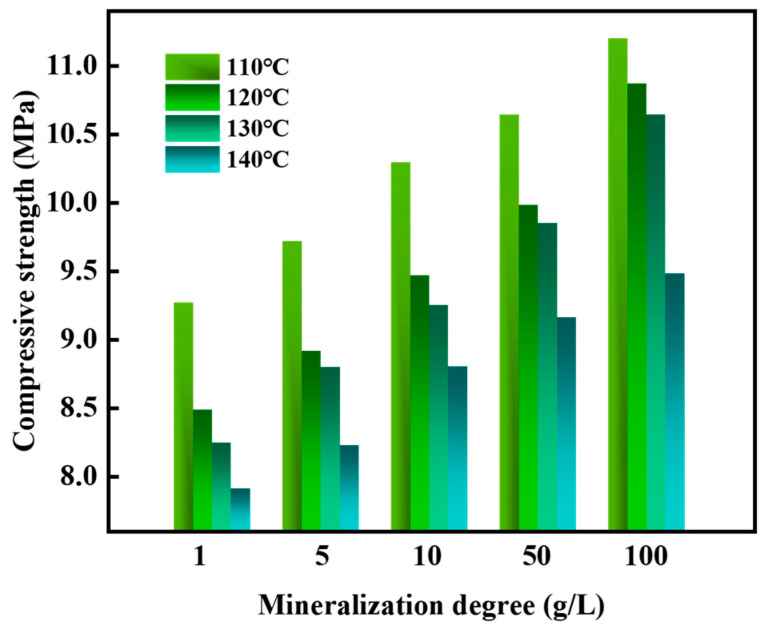
Influence of mineralization degree on the coagulation strength of the gel–resin slurry system.

The influence of different mineralization saltwater on the setting time and strength of the gel–resin composite system was studied. The results showed that the setting time of the resin was between 133 and 230 min (Figure 21), and the setting strength was between 7.7 and 11.3 MPa (Figure 22). Moreover, the higher the mineralization, the longer the setting time; the higher the temperature, the shorter the setting time.

### 2.4. The Working Performance of the Gel–Resin Coagulation Composite System

#### 2.4.1. Long-Term Stability of Gel–Resin Composite System at High Temperature

**Figure 23:**
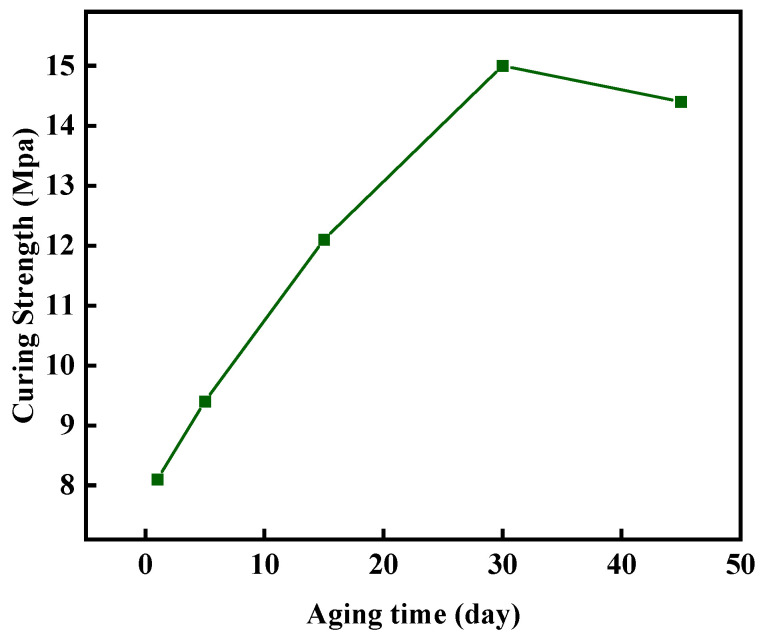
Influence of aging time on consolidation strength.

The compressive strength of the gel–resin composite system was tested after aging at 140 °C for 1 d, 5 d, 15 d, 30 d, and 45 d to study its long-term stability at a high temperature. The research results are shown in Figure 23. After aging for 45 d at 140 °C, the compressive strength of the controllable setting body can still reach 14.4 MPa, and its high-temperature stability is excellent.

#### 2.4.2. Evaluation of the Thickening Performance of the Gel–Resin Composite Coagulation System

**Figure 24:**
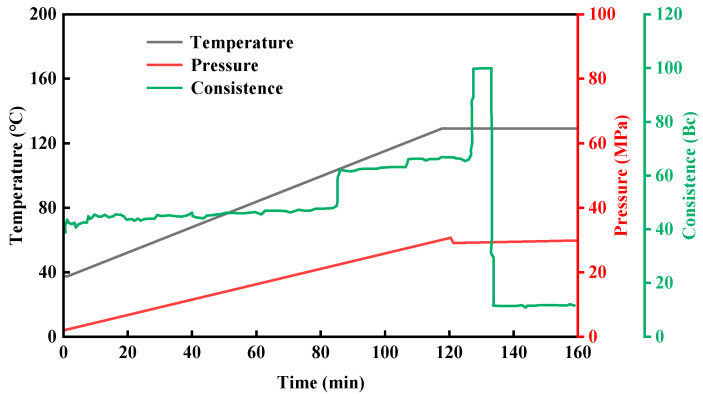
The coagulation situation of the gel–resin composite coagulation system during the thickening process.

According to GB/T 19139-2012 “Test Methods for Oil Well Cement”, the thickening performance of the resin coagulation plugging system was evaluated. The experimental [38] temperature was set at 130 °C, the thickening time at 120 min, and the pressure at 30 MPa. The experimental results are shown in Figure 24. As the test temperature and pressure increase, the consistency curve increases in a stepwise manner. When the temperature of the resin coagulation and leakage blocking system rises to 130 °C, and the pressure reaches 30 MPa, the thickening time used is 130 min, and the consistency reaches 100 Bc. As time increases, the consistency instantly drops to about 10 Bc. At this time, the resin coagulation and leakage blocking system has completely coagulated. From the graph, we can see that the resin coagulation plugging system completely coagulates in 130 min and has a certain strength. However, the thickened stirring rod does not adhere to the gel–resin composite system, which also shows that the adhesion of the resin coagulation plugging system to the drill rod is relatively small, and it will not cause difficulties in drilling and retrieving the drill rod.

### 2.5. Mechanism and Effect Evaluation of Gel–Resin Composite Coagulation System for Pressure-Bearing Leakage Plugging

#### 2.5.1. The Pressure-Bearing Plugging Ability of the Gel–Resin Composite Coagulation System in the Wedge-Shaped Crack

**Figure 25:**
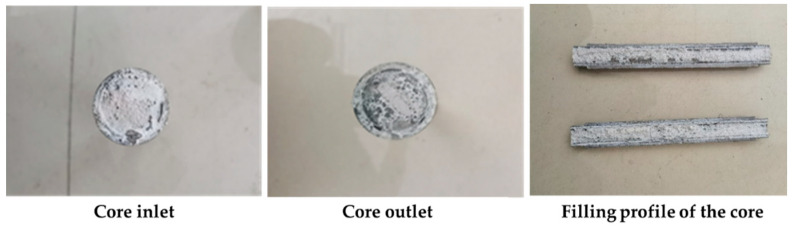
Images of filling effect of wedge-shaped crack plugging and displacement in core.

After preparing the composite gel–resin solution, it was injected into the high-temperature and high-pressure leakage plugging experimental device. The plugging effect after condensation and formation of a plug in the wedge-shaped crack with a size of 7 mm at the inlet and 5 mm at the outlet is shown in Figure 25. The composite gel–resin leakage plugging system achieved complete filling in the wedge-shaped crack, and the overall integrity of the resin plugging layer was good, which could be evenly distributed in the crack and effectively plug the crack. At the same time, it can be seen that the resin plugging agent effectively filled both the inlet and outlet of the crack, and the leakage channel was completely closed, indicating a strong plugging effect at the breakthrough pressure of the composite gel–resin leakage plugging system.

#### 2.5.2. The Pressure-Bearing Leakage-Stopping Ability of the Resin Leakage-Stopping Agent After Stepwise Pressure Testing in Cracks and Sand-Filled Tubes

**Figure 26:**
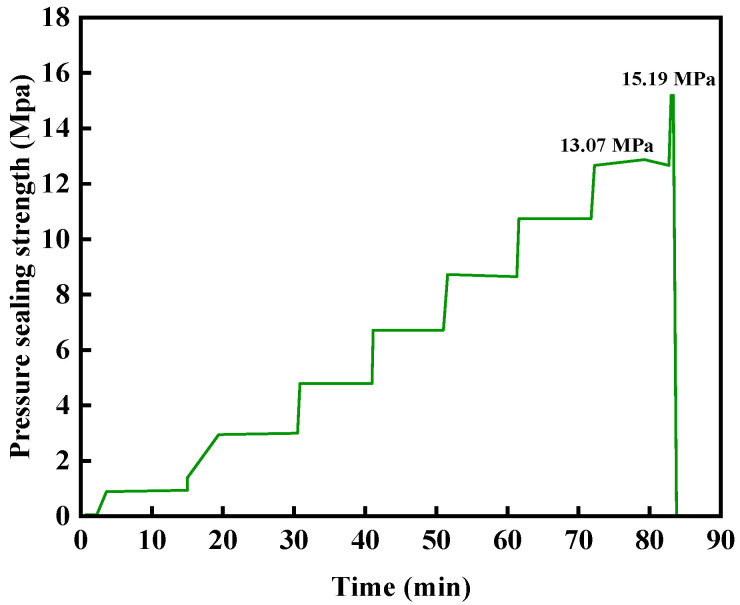
Pressure-bearing sealing capacity curve of a wedge-shaped crack (entrance 7 mm, exit 5 mm).

The pressure-bearing plugging performance of the gel–resin composite system was evaluated using a high-temperature and high-pressure plugging simulation device under the condition of 140 °C. The gelation time was 220 min at 140 °C, and the pumping rate was 10 mL/min. The plugging time was 10 min under constant pressure. The experimental results are shown in Figure 26. The results show that the resin–gelation plugging system did not leak for a wedge crack with an inlet size of 7 mm and an outlet size of 5 mm at 13.07 MPa for 10 min; it broke through after being pressurized to 15.19 MPa, which shows a good crack-plugging ability.

**Figure 27:**
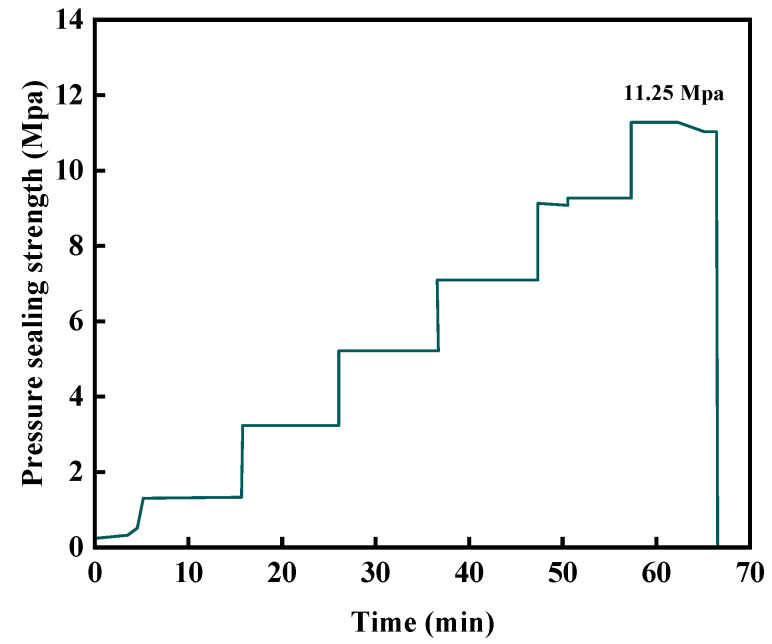
Pressure sealing capacity curve of a sand-filled tube (diameter 3.8 cm, tube length 30 cm).

The pressure-bearing sealing performance of the gel–resin composite system in the sand-filled pipe under 140 °C conditions was evaluated using a high-temperature and high-pressure sealing simulation device. Experimental conditions: 140 °C condensation for 220 min, pump injection rate of 10 mL/min, and waiting for sealing for 10 min at a constant pressure of 2 MPa. The experimental results are shown in Figure 27. The research results show that the gel–resin composite system breaks through after waiting for sealing for 7 min at a constant pressure of 11.25 MPa for the sand-filled pipe connected to the outlet 6 mm pipeline (diameter 3.8 cm, pipe length 30 cm), and it has a good sealing ability for fractures and holes.

### 2.6. The Unblocking Performance of the Gel–Resin Composite System

We investigated its plug-dissolving performance by measuring the acid solubility degradation rate of the resin condensate. The research results are shown in Table 6. Under the condition of 140 °C, we soaked the resin mortar condensate samples with 15% hydrochloric acid at a volume ratio of 1:2 and studied the acid solubility degradation of the resin mortar condensate. After soaking for 24 h, the resin mortar condensate degraded into a fluid containing a small amount of debris (filling materials), and the acid solubility degradation rate was 97.69%. Under the condition of 80 °C, we soaked the resin condensate samples with 15% hydrochloric acid at a volume ratio of 1:2 and studied the acid solubility degradation of the resin condensate. After soaking for 72 h, the resin condensate degraded into a fluid containing a small amount of debris (filler material), and the acid solubility degradation rate was 96.53%. Under the condition of 80 °C, the resin condensate sample was soaked with 20% hydrochloric acid at a volume ratio of 1:2, and the acid solubility degradation of the resin condensate was studied. After soaking for 72 h, the resin condensate degraded into a fluid containing a small amount of debris (filler material), and the acid solubility degradation rate was 97.23%. In summary, the resin condensate plugging system is easily degraded by acid, and it can be unplugged, with a low degree of damage to the reservoir.

## 3. Conclusions

(1) By optimizing the formulations of bisphenol, A epoxy resin (20%), hexamethylenetetramine (3%), and hydroxyethyl cellulose (1%) and adding nano-silica and walnut shell particle fillers, a leak-stopping system with both a controllable curing property and high strength was constructed. After aging at 140 °C for 45 days, this system still maintains a compressive strength of 14.4 MPa, with an initial decomposition temperature of 220 °C, demonstrating excellent thermal stability.
(2) When faced with contamination by foreign drilling fluids and high-salinity conditions, the resin plugging system demonstrated good anti-contamination and salt tolerance. After contamination with 20% Tarim drilling fluid, the compressive strength of the cured resin remained at 5.04 MPa. At a salinity of 100 g/L, the setting time ranged from 133 to 230 min, and the compressive strength after setting ranged from 7.7 to 11.3 MPa.
(3) The resin plugging system demonstrated an excellent performance in key aspects: (a) after aging for 45 days at 140 °C, the compressive strength of the cured resin plug reached 14.4 MPa; (b) the cured resin exhibited minimal adhesion to the drill rod; and (c) the uncured resin system showed a strong viscosity (or shear) recovery capability.
(4) The cured gel–resin plug exhibited an effective pressure sealing capability at 140 °C in a sand-packed tube test. Specifically, the plugging system was breached after 7 min under a constant differential pressure of 11.25 MPa applied across a sand-packed tube equipped with a 6 mm outlet orifice. This demonstrates its capability to effectively seal fractures and holes.
(5) Degradation tests showed that cured resin material (140 °C) mixed with 15% hydrochloric acid at a volume ratio of 1:2 (resin:acid) achieved a degradation rate of 97.69% after 24 h, indicating that the resin leakage plugging material has an excellent degradation performance.

## 4. Materials and Methods

### 4.1. Materials

The reagents and materials used for this study are shown in Table 7.

### 4.2. Rheology

The rheological properties of the resin plugging agent system were tested using a HAAKE MARS 60 rotational rheometer (Thermo Fisher Scientific (Shanghai, China) Co., Ltd.) before coagulation. In the experiment, a rotor model CC41/Ti was used (rotor diameter is 41 mm). During the test, the sample temperature needs to be balanced for at least 30 min, and the temperature error should be controlled within ±0.1 °C. To ensure the accuracy of the data, the above-mentioned rheological performance tests were all repeated three times.

### 4.3. Infrared Spectroscopy Analysis

The chemical structure of the composite gel–resin was characterized using a Fourier Transform Infrared Spectrometer (Nicolet iS50 FT-IR, Thermo Fisher Scientific (Shanghai, China) Co., Ltd.). After the sample was cleaned with deionized water to remove unreacted components, it was dried in a vacuum oven and ground into powder. The test samples were prepared using the potassium bromide pelletizing method. The test conditions were set as follows: scanning wavenumber range 4000–400 cm^−1^, ambient temperature 25 °C, resolution 1 cm^−1^, and cumulative scanning 8 times.

### 4.4. Thermogravimetric Analysis

The thermal stability of high-temperature, high-strength condensable resin powder was characterized using an American TGA550 thermal gravimetric analyzer (Waters Technology (Shanghai, China) Co., Ltd). Before the test, the resin powder was pretreated in a 105 °C oven to remove moisture. During the experiment, an accurate amount of 10–15 mg of sample was placed in a sealed crucible, and the temperature was programmatically raised from 25 °C to 600 °C at a heating rate of 20 °C/min under a nitrogen atmosphere (flow rate 50 mL/min) for testing.

### 4.5. Sealing Performance

The plugging performance of the condensable resin for cracks was studied through a high-temperature and high-pressure crack physical simulation device. The simulated crack core used in the device is a steel cylindrical structure, and the crack runs through the longitudinal section of the steel column. The dimensions of the crack are a crack length of 30 cm, crack height of 3 cm, and crack widths of 3 mm, 5 mm, 7 mm, and 10 mm, respectively. The pressure-bearing plugging capacity is one of the key parameters to measure the plugging effect. The specific steps of the leakage test are as follows: (a) adjust the temperature of the heating box to simulate the formation temperature of 140 °C; (b) place the steel fracture core with the corresponding crack width into the core holder and apply a confining pressure of 10 MPa; (c) inject the simulated drilling fluid into the fracture core at an injection rate of 10.0 mL/min until the core is saturated; (d) inject the curable resin solution into the fracture core at an injection rate of 10.0 mL/min until the resin solution completely flows out of the fracture outlet and no more water is produced; (e) seal the fracture core model and let it stand for 8 h, waiting for the resin to set as the solution completes the reaction; and (f) inject the simulated drilling fluid into the fractured rock at an injection rate of 10.0 mL/min, then record the change in injection pressure in real time through data software (version: 1.0.2). The highest pressure achieved is the pressure-bearing and leak-stopping ability of the resin when applied to the fracture.

## Figures and Tables

**Table 1:** The rheological properties of the gel–resin composite system after stirring for different times at room temperature.

Rheology	φ_600_	φ_300_	φ_200_	φ_100_	φ_6_	φ_3_	Initial/Final Shear Force	Apparent Viscosity	Plastic Viscosity	Dynamic Shear
Original state	93	61	39	24	13	8	3.5/8	46.5	32	13.9
Stirring for 5 h	99	71	41	25	16	12	9.5/9	49.6	28	20.6
Stirring for 10 h	102	88	44	26	22	16	5.5/8.8	51	14	35.4
Standing for 1 d	151	89	72	33	30	19	8/9	75.5	62	12.9

**Table 2:** Influence of different drilling fluid dosages on rheological properties of gel–resin slurry system.

Rheology	φ_600_	φ_300_	φ_200_	φ_100_	φ_6_	φ_3_	Initial/Final Shear Force	Apparent Viscosity	Plastic Viscosity	Dynamic Shear
Well slurry	18	12	10	7	2	2	6/7	9	6	2.9
Gel–resin slurry	180	123	92	59	13	9	5.5/9.5	90	57	31.6
Gel–resin paste: Well slurry 3:7	185	161	119	77	20	10	6/8	92.5	24	65.6
Gel–resin paste: Well slurry 5:5	205	153	112	67	16	13	8/11	102.5	52	48.4
Gel–resin paste: Well slurry 7:3	207	184	134	70	24	22	10/12	103.5	23	77.1

**Table 3:** Compressive strength of the gel–resin composite system treated with KCl polymeric drilling fluid.

Sample	Conditions for Condensation	Sample Diameter/mm	Maximum Force Value/N	Compressive Strength/MPa
Add 10% polymeric sulfonate drilling fluid	130 °C 150 min	36.57	6199	6.36
Add 15% polymeric sulfonate drilling fluid	130 °C 150 min	37.35	5463	5.75
Add 20% polymeric sulfonate drilling fluid	130 °C 150 min	35.53	4386	4.29

**Table 4:** Formulation of drilling fluid system for the second opening in Tarim.

Recipe Name	Material Name	Use Ratio/%
Tarim drilling fluid system	Bentonite	4.0
	Caustic soda	0.2
	Sodium carbonate	0.1
	Potassium salt of polyacrylamide	0.1
	Polymer filtration reducer	0.2
	Cationic cellulose	0.3
	Hydrolyzed polyacrylamide ammonium salt	0.5
	Sulfonated phenolic resin (dry powder)	1.0
	Lignite resin	1.0
	Potassium chloride	3.0
	Silanol inhibitor	1.0
	Asphalt anti-collapse agent	2.0
	Ultrafine calcium carbonate	1.0

**Table 5:** The compressive strength of the gel–resin composite system in Tarim drilling fluid.

Sample	Conditions for Condensation	Sample Diameter/mm	Maximum Force Value/N	Compressive Strength/MPa
Add 10% drilling fluid	130 °C 150 min	37.56	8186	7.39
Add 15% drilling fluid	130 °C 150 min	36.83	7107	6.14
Add 20% drilling fluid	130 °C 150 min	37.12	5872	5.04

**Table 6:** Acid solubility degradation rate of the gel–resin condensate.

Acid Solubility Conditions	140 °C, 24 h	80 °C, 72 h	80 °C, 72 h
Mass before acid dissolution (g)	80.09	89.34	101.42
Filling material quality (g)	8	8	8
Quality after acid dissolution (g)	9.66	10.82	10.59
Acid solubility degradation rate (%)	97.69	96.53	97.23

**Table 7:** Experimental materials.

Reagent	Parameter	Purity	Manufacturer
Bisphenol A-type epoxy resin	Epoxy value (0.43)	99.0%	Saen Chemical Technology (Shanghai, China) Co., Ltd.
Hexamethylene tetramine	\	98.0%	Saen Chemical Technology (Shanghai, Shina) Co., Ltd.
Hydroxyethyl cellulose	Molecular weight (736.7)	98.0%	Sinopharm Chemical Reagents Co., Ltd (Beijing, China).
Diethylene triamine	\	97.0%	Aladdin Biochemical Technology (Shanghai, China) Co., Ltd.
Nano-silica dioxide	Average particle (330 nm)	\	Shanghai Aladdin Biochemical Technology Co., Ltd. (Shanghai, China)
Quartz sand	Average particle (60 mesh)	\	Shandong Xiya Chemical Co., Ltd. (Binzhou, China)
Walnut shell	Average particle (3 mm)	\	Shandong Xiya Chemical Co., Ltd. (Binzhou, China)
Barite	\	\	Shandong Xiya Chemical Co., Ltd. (Binzhou, China)

## Data Availability

The original contributions presented in this study are included in the article. Further inquiries can be directed to the corresponding author.
